# The Association between ADHD and Obesity: Intriguing, Progressively More Investigated, but Still Puzzling

**DOI:** 10.3390/brainsci9100256

**Published:** 2019-09-27

**Authors:** Samuele Cortese

**Affiliations:** 1 Center for Innovation in Mental Health, School of Psychology, Faculty of Environmental and Life Sciences, University of Southampton, Southampton SO17 1BJ, UK; Samuele.Cortese@soton.ac.uk; 2 Clinical and Experimental Sciences (CNS and Psychiatry), Faculty of Medicine, University of Southampton, Southampton SO17 1BJ, UK; 3 Solent NHS Trust, Southampton SO19 8BR, UK; 4 New York University Child Study Center, New York, NY 10016, USA; 5 Division of Psychiatry and Applied Psychology, School of Medicine, University of Nottingham, Nottingham NG72UH, UK

**Keywords:** ADHD, obesity, overweight, Body Mass Index

## Abstract

This narrative review is aimed at presenting the most recent evidence on the association between attention-deficit/hyperactivity disorder (ADHD) and obesity. The review is informed by previous relevant systematic reviews and a search in Pubmed and PsycINFO up to 1 August 2019. Although the association between ADHD and obesity would seem, at first, paradoxical, in the past two decades there has been an increasing number of studies on this topic. The present review shows that there is meta-analytic evidence supporting a significant association between these two conditions, at least in adults. Growing evidence is also being published on the genetic and environmental factors underlying the association. However, the cause–effects paths, as well as the exact mechanisms explaining the association, remain unclear. Additionally, empirical evidence guiding the management/treatment of patients with the two conditions is still limited. Therefore, after almost 20 years from the first report of a link between ADHD and obesity, this association continues to be puzzling.

## 1. Introduction

Following the seminal publication, in 2002, of the study by Altfas [1], showing higher than expected rates of attention-deficit/hyperactivity disorder (ADHD) in bariatric patients with severe obesity, there has been a progressively increasing number of studies focused on the link between ADHD and obesity/overweight. In fact, a simple Pubmed search ((ADHD [ti] or Attention deficit [ti] OR Attention-Deficit [ti]) AND (obes* [ti] OR overweight [ti])) retrieved 0 hits in 2003 and 15 in 2018.

The association between ADHD and obesity may seem, at first, counterintuitive. “These children move a lot; if anything, they should be thinner compared to their non-ADHD peers” is what many lay persons or even mental health professionals may think. It goes without saying that higher than expected rates of obesity in individuals with ADHD do not rule out the possibility that underweight is also associated with ADHD, i.e., the rates of both under- and overweight may be higher in individuals with ADHD compared to those without the disorder.

Building on previous evidence synthesis in the field, this review aims to provide updated evidence on a series of aspects related to the link ADHD–obesity.

## 2. Methods

Although it is not intended as a formal systematic review with a structured qualitative and quantitative appraisal of each pertinent study, the present narrative review was rigorously informed by available systematic reviews, complemented by recent empirical quantitative studies retrieved with a search in PubMed and PsycINFO from 20 October 2017 (search date of the latest available systematic review [2]) to 1 August 2019, using the following search terms in the title or abstract, with specific syntax adapted for each of the two databases: *ADHD, Attention deficit, Attention-Deficit, Hyperkinetic Disorder, obesity, overweight*. For ease of use, the present review is organized in a question and answer format. Any retrieved study (systematic review or individual empirical study) pertinent to the review questions was retained for the present review.

## 3. What Is the Evidence Supporting a Significant Association between ADHD and Obesity?

To date, two systematic reviews [3,4] with meta-analyses aimed at estimating the association between ADHD and obesity/overweight are available. Even though they have been published almost simultaneously and are quite similar in a number of methodological aspects, they also differ in terms of some of the inclusion criteria and, hence, of their findings.

In the first of the two systematic reviews/meta-analyses, Cortese et al. [3] included observational (population-based or clinical) studies reporting the degree of association (or data allowing to estimate it) between ADHD and obesity. ADHD was defined as per DSM/ICD criteria, on the basis of self-report, severity score above a cut-off threshold on a validated scale, or diagnosis available in medical registries. Obesity was defined by the presence of a body mass index (BMI) ≥30 in adults and any available definition in children. Studies in bariatric clinics were excluded, to avoid a possible selection bias. Unpublished data were systematically gathered from a number of authors in the field. After pooling data from 41 studies (including a total of 48,161 individuals with ADHD and 679,975 comparisons), a significant association between obesity and ADHD was found for both children (crude odds ratio, OR = 1.20, 95% CI = 1.05–1.37) and adults (crude OR = 1.55, 95% CI = 1.32–1.81). This translated in an increased pooled prevalence of obesity by about 70% in adults with ADHD (28.2%, 95% CI = 22.8–34.4) compared with those without ADHD (16.4%, 95% CI = 13.4–19.9), and by about 40% in children with ADHD (10.3%, 95% CI = 7.9–13.3) compared with those without ADHD (7.4%, 95% CI = 5.4–10.1). Of note, the association hold significant even when odds ratios adjusted for possible confounding factors were pooled in the meta-analysis (aOR = 1.27, 95% CI = 1.11–1.44). Furthermore, results remained significant in a series of sensitivity analyses limited to studies where the diagnosing of ADHD was made via direct interviews (the most rigorous diagnostic approach) or using directly measured height and weight (as opposed to the less accurate self-reported data). ADHD resulted also significantly associated with overweight (defined as BMI ≥ 25 in adults or ≥ 85th percentile in children). Additionally, meta-regression analyses showed that sex, study setting, study country, and study quality did not significantly impact on the association between obesity and ADHD. Of note, the high statistical heterogeneity found in the primary analysis generally tended to decrease in the sensitivity analyses that included more homogenous clinical groups. The only analysis in which the association was not significant was the one limited to studies (*n* = 12) where all individuals with ADHD were medicated with stimulant medications.

The second systematic review/meta-analysis by Nigg and colleagues [4] included studies with similar criteria in terms of definition of ADHD but pooled also studies in which an association between BMI values (as dimensional scores, rather than obesity defined as a category) and ADHD was provided. Additionally, there is no evidence, from the published text of the paper, that the authors included unpublished data. After pooling 43 studies (including a total of 703,937 individuals) Nigg et al. found a pooled OR = 1.22 (95% CI: 1.11–1.34). When restricting the analyses to studies that controlled for stimulant medication use, the pooled OR was still significant (1.30, 1.12–1.50). Considering sex effects, the association was significant in females (1.19, 1.01–1.41) but not in males (1.10, 0.95–1.23). Moreover, when focusing on the effect of age, the association was larger in adults (1.37 1.19–1.58) than in children/adolescents (1.13, 1.00–1.27). No measures of heterogeneity were reported by Nigg and colleagues.

Overall, while these two meta-analyses (from two independent groups) substantially agree on the effect of the association between ADHD and obesity when pooling studies across ages and in both sexes, they diverge in terms of the estimation of age and sex effects. However, they both agree that the relationship between ADHD and obesity is stronger in adults compared to children/adolescents. Interestingly, sex and ADHD presentation effects have been found in an additional study published after these two meta-analyses [5]. Drawing on data from the National Longitudinal Survey of Adolescent to Adult Health, Waves II to IV (including 13,332 participants, aged 12–34 years), Inoue et al. reported that, compared with individuals without ADHD, both males and females with ADHD hyperactive impulsive presentation (as per DSM-5 terminology) had significantly higher BMI, whereas the ADHD inattentive presentation was associated with higher BMI in females only.

The two meta-analyses could not explore in detail the effect of psychiatric comorbidities associated with ADHD. In this regard, a recent study [6] found that, compared to the general population, the incidence of obesity was significantly higher in patients with ADHD and comorbid adjustment disorder (22.22% vs. 4.42%) or learning disability (19.05% vs. 4.42%). This study suggests the need to accurately define the phenotype of individuals with ADHD enrolled in studies aimed to explore the link ADHD–obesity, taking comorbidities into account.

## 4. Does ADHD Contribute to Obesity/Overweight or Is It the Opposite?

The two systematic reviews/meta-analyses described in the previous section used cross-sectional data. As such, they are not informative on temporal and cause–effect relationships. Indeed, it is possible that: (1) ADHD precedes and contributes to obesity, deregulating energy balance; (2) Obesity leads to ADHD symptoms; (3) The two disorders share common etiopathophysiological mechanisms; (4) There are bi-directional links between the two conditions, with, possibly, additional underlying common factors contributing to the association. A few studies attempting to address these issues are available to date. In the first, Cortese et al. [7] analysed data from a US longitudinal clinical cohort of individuals diagnosed with (the equivalent of) ADHD in the early childhood and followed prospectively for 33 years (mean age at last follow-up: 41 years). Individuals with childhood ADHD had significantly higher BMI (30.1 ± 6.3 vs. 27.6 ± 3.9) and obesity rates (41.4% vs. 21.6%) than those without childhood ADHD, even after adjusting for socioeconomic status and lifetime mental disorders. Furthermore, participants with persistent and remitted ADHD did not differ significantly in BMI or obesity rates. After adjustment, individuals with remitted, but not persistent, ADHD had significantly higher BMI and obesity rates compared to those without childhood ADHD. However, as usable data on baseline weight and height were not available, despite using information from a longitudinal study, Cortese et al. were not able to firmly establish a temporal and cause–effect relationship between ADHD and obesity. The study by Khalife et al. [8] provided more insight on the temporal link. The authors followed a sample of 7- to 8-year-old children (*n* = 8106) until the age of 16, when data relevant for the study were available for 6934 participants. They found that teacher-reported ADHD symptoms in childhood significantly predicted adolescent obesity (based on parental reports of weight and height), rather than vice versa. More specifically, symptoms of inattention and hyperactivity at the age of 8 were significantly associated with obesity at 16 years of age, even after adjusting for sex, baseline BMI, physical activity, family structure change, and maternal education. The lack of a formal diagnosis of ADHD, as well as of direct measures of height and weight, are limitations that should be considered when interpreting the study results. A more recent study [9] partially addressed these limitations. The authors followed up 336 children with a research-based diagnosis of ADHD and 665 age- and sex-matched non-ADHD controls. Compared to the controls, during the follow-up, cases with ADHD were 1.23 (95% CI, 1.00–1.50) times more likely to present with obesity, defined on the basis of weight and height retrospectively extracted from medical records. The retrospective nature of the ADHD and obesity assessment, as well as the lack of adjustment for a number of variables, including anxiety, depression, eating disorders and substance abuse, were study limitations acknowledged by the authors themselves. Another recent study [10] concurred with the previous findings, reporting that, in a sample of 3903 children form the R cohort, clinically significant scores of ADHD at the age of 6 predicted a significant increase in fat mass (rigorously measured with energy x-ray absorptiometry) in the following three years, regardless of possible alterations in eating patterns, while there was no evidence of a reverse association. Whilst the previous studies converge in suggesting that ADHD precedes, and, hence, possibly causally contributes to obesity, a recent study challenged this view. Martins-Silva et al. [11] used a bi-directional two-sample Mendelian randomisation design on aggregate data from consortia of genome-wide association studies to assess if ADHD had a causal effect on BMI increase in children (*n* = 35,668) as well as adults *(n* = 322,154–500,000). To this end, the authors used the inverse weighted variance (IVW) estimator, i.e., a linear regression of the instrument–outcome association estimates on the instrument–exposure association estimates, weighted by the inverse of the variance of the instrument–outcome association estimates. The authors found evidence indicating a positive causal effect of BMI on ADHD, rather than the opposite. The conclusion of this study should be considered in the light of the study limitations, as, whilst 77 variants were available to test the effects of BMI, only 12 variants were used to assess the effects of ADHD. Furthermore, the study used a dimensional approach, as its focus was on BMI rather than obesity/overweight; as such, the study conclusions are not comparable to those of the other studies cited in this section, which used a categorical approach (presence or absence of obesity/overweight). Whilst the mechanisms leading from obesity to ADHD may not seem straightforward to understand, it has been hypothesized that obesity may lead to ADHD symptoms via sleep disordered breathing (SDB). In fact, SDB is associated with obesity and has been shown to lead to ADHD-like symptoms [12]. Furthermore, anecdotal reports note individuals with obesity and bulimic or abnormal eating behaviors may present with repeated and impulsive interruptions of their activities with the aim to get food, resulting in ADHD symptoms such as disorganization, inattention, and restlessness [12]. Additionally, as discussed more in depth below, obesity is associated with a chronic inflammatory status, which could induce ADHD symptoms. Admittedly, these are speculative explanations that need rigorous testing.

Overall, considering all the available studies, it is fair to conclude the direction of the causal associations between ADHD and obesity is far from being clear, and it is likely that complex bidirectional associations, rather than simple unidirectional ones, are involved.

## 5. What Are the Factors Underpinning the Association between ADHD and Obesity?

A review informed by a systematic search performed in October 2017 [2] discussed the putative mechanisms underlying the link between ADHD and obesity assessed so far. Here, the mechanisms discussed in that review are summarised, complemented by evidence from recent relevant studies published after October 2017.

### 5.1. Genetic Factors

Whilst early reviews [12,13] on the topic put forward the hypothesis of common genetic pathways but could not back up this with solid empirical evidence, large-scale studies published in the past few years have supported the role of genetics.

In a large study based on studies registries, Chen et al. [14] observed that siblings of index males with overweight/obesity (in total, *n* = 472,735) had increased risk of ADHD (obesity: OR = 1.42, 95% CI = 1.24–1.63; overweight: OR = 1.14, 95% CI = 1.05–1.24;) compared with siblings of index males without obesity, after adjusting for birth year of the index male and sex of the sibling. The results remained significant even after adjusting for the ADHD status of the index male (obesity: OR = 1.38, 95% CI = 1.21–1.57; overweight: OR = 1.13, 95% CI = 1.04–1.22). This study showed a familial co-aggregation of ADHD and obesity but not necessarily a common genetic cause, as “familial” does not equal “genetic”. Likewise, another study from an independent Dutch sample [15] based on 1828 youth assessments from 447 unique families showed no significant difference in the risk of obesity between youth with ADHD and their unaffected siblings. However, compared to the expected prevalence, all ADHD family members (index cases with ADHD, siblings without ADHD, mother and father) had increased risk of overweight. The study found that parental overweight, rather than parental ADHD, was predictive of offspring overweight. The authors concluded that shared unhealthy lifestyle factors (such as nutrition, sleep, exercise, stress) as well as genetic factors shared by family members, are likely to explain the link between ADHD and obesity/overweight. Therefore, according to this study, there would be a common cause in the family environment rather than a causal relationship between ADHD and overweight.

In contrast, another large Swedish registry study [16] (2,538,127 individuals born between 1973 and 2000) showed that cases with ADHD were at an increased risk of clinical obesity compared with controls without ADHD. Interestingly, the strength of the co-aggregation decreased with decreasing genetic relatedness (from siblings to cousins). The correlation between the vulnerability to ADHD and clinical obesity was deemed to be entirely attributable to genetic correlation (rg 0.30, 95% CI 0.17–0.44). Of note, in a recent genome-wide meta-analysis of 20,183 individuals diagnosed with ADHD and 35,191 controls, a genetic correlation of 0.26 between ADHD and BMI was found [17].

Overall, considering these mixed findings, it seems that a simple but naïve dichotomy between gene and environment should be avoided, and it should be acknowledged that the link between obesity and ADHD is likely explained by genetic alterations which contribute to an obesogenic environment, as well as by behaviours that lead to weight gain and interact with environmental factors, which will be detailed in the next sub-sections.

Interestingly, recent evidence pointed to possible sex effects. In a sibling pairs study, Do and colleagues [18] found that unique or non-shared environmental influences contributed to the relationship between ADHD and obesity in males, whilst genetic factors contributed to the relationship in females. The reasons for this dissociation deserve further elucidation.

Groundbreaking research started elucidating the links between common genetic underpinnings and neuronal correlates of the association ADHD–obesity. Drawing on data from the IMAGEN sample, Barker et al. [19] found not only that ADHD symptoms, and, more specifically, impulsivity (measured at age 19) of 874 participants were significantly associated with their BMI, but also that there was a significant correlation between the polygenic risk scores for impulsivity and BMI. Furthermore, both polygenic scores were significantly associated with a neuronal endophenotype located bilaterally in the cerebellum, amygdala, hippocampus, para-hippocampus and orbital frontal cortex, and the in left infero-temporal cortex. The authors concluded that this common neuronal substrate may, at least in part, underpin the shared genetic liability for ADHD and BMI. These findings should be interpreted in the light of the fact that, as the authors noted, generally PRS explain a small proportion of the vulnerability to mental health illness. Additionally, these findings should be replicated in clinical samples.

### 5.2. Positive Energy Balance: Abnormal Eating Patterns and Executive Functions

A positive energy balance is achieved when the amount of energy accumulated in the body is higher than the amount of energy that is expended. This can result from overeating but also from decreased physical activity levels and sedentary lifestyle. As reviewed in [2], there is evidence that abnormal and dysfunctional eating patterns are more frequent in ADHD compared to non-ADHD controls. These abnormal eating patterns include binge eating/bulimic behaviours (for instance [20]), skipping breakfast, eating in the evening and at night, eating more calorific food/snack/junk food, and overeating in response to negative mood. However, evidence is somehow conflicting, and the specific eating alterations remain unclear. For instance, Egbert et al. [21] found that ADHD symptoms were significantly associated with objective binge eating (with loss of control) but not with subjective binge eating. ADHD symptoms have also been found related to body image dissatisfaction, which may mediate the association between ADHD and disordered eating, leading to overweight. A moderation model, including age, family income, child BMI z-score, and parent BMI as covariates, found a significant interaction between ADHD symptoms and body dissatisfaction, with the overall moderation model explaining 16.26% of the variance in disordered eating attitudes and behaviours.

Moving beyond the behavioural level, by using structural equation modelling in a sample of 421 non-clinical adults, a recent study aimed to gain insight into the genetic mechanisms underpinning ADHD and indices of overeating by genotyping Dopamine receptor D4 and dopamine receptor D2 (DRD4 and DRD2), considering their involvement in ADHD and reward sensitivity [22]. It was found that participants with high levels of ADHD symptoms and genetic profiles associated with greater dopaminergic activation in key brain reward areas were more likely to engage in hedonic eating and, as a result, have a higher BMI.

Another study added to our knowledge on abnormal reward mechanisms, providing a different perspective. In a sample of 25 individuals (aged 17–68 years) with obesity and 25 with normal weight, van der Oord et al. [23] found that those with binge eating, as determined by their score on the Eating Disorder Examination Questionnaire (EDEQ) [24] binge eating item, scored significantly higher on Delay Discounting and the ADHD inattention scale compared to those without binge eating, even though results were no more significant after controlling for inattention symptoms. Overall, these findings point to shared mechanisms towards both ADHD and obesity with binge eating. Of note, abnormal reward mechanisms are part of the so-called hot executive dysfunctions that involve incentives and motivation and are underpinned by the paralimbic orbito-medial and ventromedial fronto-limbic structures [25]. Alterations in hot executive functions have been found in patients with obesity [26]. Whilst it was unexpected that, compared to those without binge eating, individuals with obesity and binge eating did not differ in the impulsivity/hyperactivity symptoms, it should be considered that the sample size was not powered to firmly test these hypotheses.

Another study set out to test, in a sample of 109 boys with ADHD (7–17 years), the relationship between overweight, polymorphism in selected genes, and cold executive dysfunctions expected to lead to abnormal eating patterns via impulsivity and inattention [27]. Tests used to investigate the executive functions included: Continuous Performance Test, Stroop Colour–Word Interference Test, Trail Making Test A and B, Matching Familiar Figures Test, Verbal Fluency Test, Rey–Osterrieth Complex Figure Test and Wisconsin Card Sorting Test. The authors reported that overweight in boys with ADHD was associated with polymorphisms in three candidate genes (*DRD4, SNAP25* and *5HTR2A*), but no significant differences in the scores of the neuropsychological tests were detected between patients with overweight and those without overweight. This study challenged the hypotheses from pervious reviews [12,13], suggesting that abnormal dopaminergic function might lead to abnormal eating habits and, in turns, to overweight, although one should consider the limited power of the sample to detect small effects.

A recent study has focused on eating behaviours alterations other than binge eating, exploring the relationship between food addition and ADHD symptoms in individuals with obesity. Brunault et al. [28] included 105 patients seen for obesity in a tertiary level setting. They found that self-reported food addiction, food addiction scores on the Yale Food Addiction Scale 2, and binge eating scores on the Binge Eating Scale were all associated with diagnoses of ADHD, with a larger effect in adults than in children. The strengths of the study (in particular, the use of tools to establish formal diagnoses of ADHD, rather than relying on symptoms severity/count only) should be considered alongside the possible study limitations, including the relatively small sample size.

In sum, the exact relationships between specific abnormal food behaviours and executive dysfunctions overweight in ADHD have not been fully elucidated and should prompt further research using well-powered samples.

### 5.3. Positive Energy Balance: Decreased Physical Activity and Sedentary Lifestyle

The review by Hanć and Cortese [2] reported evidence, deriving mostly from population-based studies, of decreased energy expenditure in ADHD due to longer exposure time to television/videogames and decreased levels of physical activity. An additional study published after this review has confirmed these data. Drawing on data from the 2011 National Survey of Child Health, Tandon and colleagues [29] showed that children with ADHD failed to engage in the amounts of physical activity, sleep, and screen time recommended for their age. There was even a significant difference between ADHD and other chronic somatic conditions, such as asthma, with children with ADHD showing 50% lower odds of sports participation than children with asthma. However, a further study [30] conducted in the Republic of Korea on 352 children (5th–6th grades) and based on parental reports has challenged this notion, finding no evidence of a significant effect of screen time on the link between ADHD and obesity. Given the importance of these data in terms of supporting a prevention strategy, additional research should be conducted to understand possible differences across countries and settings.

### 5.4. Sleep Patterns Alterations

An intriguing line of research has pointed to a possible role of disrupted sleep patterns. This is based on the premise that excessive daytime sleepiness (EDS), caused by sleep disturbance or manifesting itself as a primary disorder, contributes to ADHD symptoms [31]. Indeed, according to the “hypoarousal theory’’ of ADHD initially formulated by Weinberg and Brumback [32], subjects with ADHD symptoms (or at least a subgroup of them) might actually be sleepier than controls, and their hyperactivity and impulsivity would be aimed to stay awake and alert, to counteract the tendency to fall asleep. Interestingly, individual with obesity have been found to present with higher than expected rates of EDS, which may be due to sleep disordered breathing or to metabolic and/or circadian abnormalities [33].

Whilst at the time of the review by Hanć and Cortese [2] only two studies [34,35] were available, one supporting and another one challenging the “sleep hypothesis” in the link, a recent study, published after the review, has provided further support to the hypothesis. Türkoğlu and Çetin [36] conducted the first study that investigated the possible role of alterations in chronotype in mediating the association between ADHD and obesity. In fact, disruption of circadian rhythms may lead to daytime sleepiness, as it entails a shift in the usual sleep patterns which may be incompatible with societal norms. Indeed, persons with a tendency to fall asleep and wake up much later (i.e., eveningness types) than those who tend to wake up and go to bed early (morningness types) may experience EDS when forced by societal norms to wake up earlier than their desired schedule. In a sample of 110 children/adolescents (7–17 years) with ADHD, Türkoğlu and Çetin found that morningness preference was 86.84% in individuals with normal BMI vs. 26.19% in those with BMI indicating obesity. Additionally, eveningness preference was found in 7.89% of those in the normal BMI and in 61.90% of participants with obesity. Further studies, ideally including objective measures of melatonin dim light onset, would be suitable to gain insight into this intriguing topic.

### 5.5. Inflammatory Mechanisms

A previous review [13] hypothesized a role of inflammation in the link between ADHD and obesity. In fact, obesity is characterised by chronic inflammation, and inflammatory processes have been shown to impact on brain functions including attention and other executive functions [37]. Therefore, the hypothesis is that obesity would induce ADHD symptoms via chronic inflammation.

While until recently there was no empirical evidence in relation to this, a recent pilot study provided initial evidence supporting a possible role of inflammatory mechanisms. In this study of 52 children seen in an obesity clinic, Cortese et al. [38] found a significant correlation between cytokines involved in inflammatory processes, i.e., IL-6 and TNF-alpha, and severity of hyperactivity/impulsivity symptoms rated by both parents and teachers. This small pilot study sets the ground for larger studies ideally aiming to longitudinally follow the impact of early inflammatory alterations, linked to obesity, on later neurocognitive aspects.

## 6. Is There Evidence of a Possible Involvement of Other Neurobiological Mechanisms?

To date, evidence fails to support a role of abnormal thyroid hormones in the link between ADHD and obesity. In fact, in a relatively large study [39] (including 26 overweight children with ADHD, 56 normal-weight children with ADHD, 66 overweight children without ADHD and 82 normal-weight children without ADHD), the concentrations of thyroid parameters (TSH, fT3 and fT4) were not significantly different in overweight children with ADHD compared to overweight children without ADHD.

A recent small study [40] found significantly lower plasma levels of adiponectin and significantly higher levels of the leptin/adiponectin (L/A) ratio in the ADHD group (*n* = 36) compared with healthy controls (*n* = 40), suggesting that a low adiponectin level and high L/A may underlie the link ADHD–obesity. Unfortunately, the cross-sectional and correlational nature of the study prevents any causal inference to be drawn.

## 7. Which Are the Implications of the Link ADHD–Obesity for the Treatment/Management of Patients with Both Conditions?

A previous review by Cortese and Castellanos [41] noted that the significant association between ADHD and obesity has important implications for the clinical management of these two conditions when co-existing but at the same time noted the paucity of evidence from primary, empirical studies to inform these clinically relevant aspects. More specifically, this review cited two studies showing how treating ADHD or dysfunctions related to ADHD may improve the outcome of obesity. In the first study, Levy et al. [42] reported that, in a group of 242 adult patients with a lengthy history of weight loss failure, those treated with stimulants following the detection of previously undiagnosed ADHD lost 12.36% of their initial weight, whereas those with no diagnosis of ADHD (and, hence, no treatment) gained, on average, 2.78% of their initial weight. Whilst one may argue that the weight loss was due to the anorexigenic effects of the stimulants, the authors noticed that it disappeared in general after two months, while the benefits on weight loss were noted at the follow-up on average at 466 days. The authors argue that these effects may be accounted for by the improvement in executive functions (attention, organisation, planning, etc.) which in turns favoured a better adherence to the weight loss programme. The conclusion of the Levy et al. study are in line with the findings from another more recent clinical study [43] highlighting how symptoms of ADHD were associated with an attenuation of weight loss following bariatric surgery in 30 adults with severe obesity, accounted for by deficit in emotional regulation. Interestingly, the Levy et al. study also resonates with the findings of a recent meta-analysis [44], showing that, compared to patients without ADHD, those with ADHD presented with a statistically significant reduction in postoperative follow-up (poor adherence to follow-up visits). Although the results of the study by Levy et al. and the most recent studies are potentially highly relevant, caution should be used when interpreting them, as they are not based on randomised evidence. Likewise, the results of another recent study by Karbasi Amel et al. [45], showing the positive effects of cognitive behavioural therapy in terms of decreasing ADHD symptoms severity and BMI as well as increasing self- esteem, should be taken with caution, given the lack of randomisation.

However, a randomised study highlighted the value of treating executive dysfunctions (which are related, albeit not consistently, to ADHD) to improve outcomes during weight loss programmes. Verbeken and colleagues [46] randomised 44 children (8–14 years of age) within the framework of a weight loss programme to 1) a six-week computerised executive function training or 2) a standard weight-control management. After 8 weeks of training, patients assigned to the executive function training group showed significantly better weight loss maintenance than those in the control group. The enthusiasm from this finding should be tempered by the fact that effects became non-significant after 12 weeks of follow-up, which suggests the possible need for cognitive training booster sessions to preserve the treatment effects.

Whilst no other similar randomised studies were found, the updated search retrieved the protocol of a randomised trial [47] aimed to assess the effects of bright light therapy versus physical exercise to prevent the occurenc of co-morbid depression and obesity in adolescents and youth with ADHD. Given the clinical relevance of these conditions and the methodological rigour with which the study was designed, it will arguably attract significant interest in the field.

Another aspect which is related to treatment/management issues is around the specific effects of stimulants (the most efficacious medications for ADHD core symptoms, at least in the short term [48]) in patients with ADHD and obesity. Anecdotally, practitioners report that, whilst some clients lose significant weight with methylphenidate over a short period of time, for others the effects are minimal. Unfortunately, empirical evidence so far does not seem to provide straightforward conclusions. Indeed, if a recent non-randomised study of 724 children [49] found higher weight decrease in obese, compared to non-obese, children with ADHD following stimulant treatment (1–3 years), in another case–control study [9], the rates of obesity were not significantly different between treated and non-treated individuals with ADHD.

Overall, it appears that available evidence is still insufficient to rigorously guide the practice of clinicians treating the challenging subgroup of patients with ADHD presenting with co-occurring obesity. It is hoped that more rigorous randomised study designs, ideally based on pragmatic RCTs, will be used in the near future.

## 8. Conclusions

After almost 20 years from the first study pointing to a significant association between ADHD and obesity, the association is now well established, at least in adults, on the basis of meta-analytic findings, and the underpinning causes are being progressively more investigated, although aspects related to the cause–effect links, the exact underpinning mechanisms and how treatment of ADHD impacts on obesity outcomes remain unclear. Additionally, the specific aspects of ADHD (core behavioural symptoms or associated neuropsychological dysfunctions) related to obesity should be further investigated.

Researchers in the field should aim to build a comprehensive biopsychosocial model that takes into account the developmental perspective to gain insight into association between ADHD and obesity. Prospective longitudinal studies, ideally including putative biomarkers, would be needed to address this from a rigorous methodological standpoint.

It is also expected that the future research in this field will provide more rigorous evidence to inform clinical decision-making when treating patients with co-existing ADHD and obesity. Ideally, this line of research should rely on pragmatic randomised controlled trials, aimed at rigorously testing the possible incremental befits in screening and treating ADHD in patients with obesity.

It is also hoped that researchers will take a less restrictive approaches and, rather than focusing on the relation between ADHD and, selectively, obesity, they will analyse this link within the framework of a broader association of ADHD with a number of somatic conditions. In fact, ADHD has been associated with other somatic conditions, such as asthma [50] (for a comprehensive overview, the reader is invited to read the paper by Instanes and colleagues [51]). Taking a broader perspective will allow us to understand more accurately possible underlying mechanism (such as inflammatory mechanisms, involved, for instance, in both asthma and obesity) and arguably reflect on the clinical reality where patients with ADHD, especially adults, struggle with more than one somatic condition.

Ultimately, all these lines of research should contribute to provide the evidence base for a more comprehensive care of patients with ADHD.
